# Identification of Tea Storage Times by Linear Discrimination Analysis and Back-Propagation Neural Network Techniques Based on the Eigenvalues of Principal Components Analysis of E-Nose Sensor Signals

**DOI:** 10.3390/s91008073

**Published:** 2009-10-14

**Authors:** Huichun Yu, Yongwei Wang, Jun Wang

**Affiliations:** 1 Department of Biosystems Engineering, Zhejiang University, 268 Kaixuan Road, Hangzhou 310029, China; E-Mails: wjzyzwz@163.com (H.C.Y.); jwang@zju.edu.cn (J.W.); 2 Food and Bioengineering Department, Henan University of Science and Technology, 48 Xiyuan Road, Luoyang 471001, China

**Keywords:** tea, electronic nose, principle components analysis, linear discrimination analysis, BP-neural network, storage time

## Abstract

An electronic nose (E-nose) was employed to detect the aroma of green tea after different storage times. Longjing green tea dry leaves, beverages and residues were detected with an E-nose, respectively. In order to decrease the data dimensionality and optimize the feature vector, the E-nose sensor response data were analyzed by principal components analysis (PCA) and the five main principal components values were extracted as the input for the discrimination analysis. The storage time (0, 60, 120, 180 and 240 days) was better discriminated by linear discrimination analysis (LDA) and was predicted by the back-propagation neural network (BPNN) method. The results showed that the discrimination and testing results based on the tea leaves were better than those based on tea beverages and tea residues. The mean errors of the tea leaf data were 9, 2.73, 3.93, 6.33 and 6.8 days, respectively.

## 1. Introduction

Tea is the one of the most widely consumed drinks in the World because of its health-giving, dietetic and even therapeutic qualities. There are numerous beneficial components in green tea, such as vitamins, catechins, amino acids, and many inorganic nutritional components. In particular, green tea antioxidants may have a protective role in inhibiting cancer [1]. However, the storage time could affect the quality of the tea, which could decrease and many of the beneficial components could be gradually lost during storage. When the quality of the tea decreases and beneficial components are lost, the aroma of the green tea will change.

Aroma plays an important role in the evaluation of tea quality. Commonly, the aroma and the taste, which are main indicators of the quality of a tea, are affinitive and interact each other, so aroma is an important index for the quality evaluation of tea. Tea can lose its aroma gradually during storage because of the influence of the temperature, humidity, sunrays and oxidation. When the storage time is longer, the aroma becomes fainter and the tea quality is inferior, so tea quality can be evaluated by detecting the changes of its aroma during storage.

Usually, tea quality is evaluated by a human taste panel, but these sensory test results can be affected by subjective factors such as emotion, exhaustion and physiological conditions. The E-nose is an increasingly fast, reliable and non-destructive technology, which can be made easy-to-use and cost-efficient, and many positive applications of the E-nose technology have been reported in numerous fields [2–4]. For example, E-noses have been used to evaluate the quality of modified atmosphere packaged poultry meat [5], spoiled beef [6], fish [7], milk [8] and apricot cultivars [9]. An E-nose was used to discriminate four types of red wines which were made from the same variety of grapes and came from the same cellar [10]. Successful discrimination by an E-nose of different Spanish wines made from different grapes was reported too [11].

There is some literature on the application of E-noses in the discrimination of different types of teas [12,13]. The E-noses were used to classify tea samples that had undergone different processing methods. In such cases, a very good sensitivity to the tea aroma and satisfactory time stability was observed for metal oxide semiconductor (MOS) sensors. Moreover, the discriminated analytical methods were discussed and reported. Recently, Bhattacharyya *et al.* monitored the volatile components of black tea during the fermentation process and detected the optimum fermentation time on the basis of peaks in the sensor outputs [14]. In his other paper, a new E-nose-based approach for monitoring tea aroma during fermentation is proposed. Two methods, namely the 2-Norm method (2NM) and the Mahalanobis distance method (MDM) were tested and the results were correlated with the results of colorimetric tests and human expert evaluation [15].

In the research related to E-noses, the datasets were analyzed by pattern recognition methods, but little detailed information is available on the pretreatment of the data obtained by the E-nose. In this paper, in order to decrease the data dimensionality and optimize the results the vector principal component analysis (PCA) method was employed for data pretreatment. The five main principal components values were extracted and used as the input of the LDA and BP neural network studies to examine the applicability of an E-nose for assessment of the storage time of the tea.

## 2. Materials and Methods

### 2.1. Electronic Nose and Data Acquisition

The experimental instrument was a portable electronic nose (E-nose, PEN2) provided by WNA Airsense Analysentechnik GmbH (Schwerin, Germany). The device was equipped with 10 different metal oxide sensors positioned in a small chamber. The E-nose system consisted of a sampling apparatus, a detector unit containing the array of sensors, and pattern recognition software for data recording and analysis. The used sensors and their main attributes were described in our previous reports [4,16–18].

During the measurement process the headspace gas was pumped into the sensor chamber with a constant rate of 100 mL/min via Teflon-tubing connected to a needle. When the gas accumulated in the headspace of vials was pumped into the sensor chamber, the ratio of conductance of each sensor changed. The sensor response was expressed as the ratio of conductance (G/G0) (G and G0, the conductivity of the sensors when the sample gas or zero gas blows over). The measurement procedure was controlled by a computer program. The measurement phase lasted for 60 s, which was enough for the sensors to reach stable values. The interval for data collection was 1 s. A computer recorded the response of the E-nose. When the measurement was completed, the acquired data was properly stored for later use and a cleaning phase lasting 70 s to clean the circuit and return sensors to their baseline values began.

### 2.2. Experimental Samples and Storage of the Tea

Longjing green tea (AAA grade, ¥ 2,400/kg in international trade) was produced and obtained on 1-Jul-05 from the Tea Academy of Zhejiang University. The tea samples were sealed in small tin bags, each of which contained 5 g tea. Two hundred and twenty five tea packages were prepared and 45 packages tea were detected in each experiment. The first 45 samples were detected on 1-Jul-05; others were kept under cold storage in the refrigerator at 4 °C. The second 45 packages were taken out from the refrigerator and detected after two months (1/9/2005), the third detection was carried out after four months (1/11/2005), the fourth detection was performed after six months (1/1/2006) and the fifth detection was performed after eight months (1/3/2006).

### 2.3. Experimental Method

#### 2.3.1. Tea Leaves Testing Sample Preparation

During this experiment 45 packages of tea samples were taken from the refrigerator and placed into 45 vials (500 mL), which were tightly sealed for 45 min. All the detections were carrying out at a constant temperature of 25 ± 1 °C. Headspace gas was pumped into the sensor chamber of the E-nose.

#### 2.3.2. Tea Beverage and the Tea Residue Testing Sample Preparation

After the tea leaves samples were detected, they were then brewed based on the criteria of the sensory panel assessment (SB/T 10157-93) [19]. Five g of tea leaves was brewed with 250 mL boiled table-water (the ratio of the tea leaves to water was 1:50), and the tea beverage was filtered after 5 min. The tea beverages and tea residues were separated, sealed in 500 mL vials and maintained for 45 min. The tea beverages were cooled to a temperature of 25 ± 1 °C during the headspace generation time. In order to protect the sensors, silica gel was used to absorb the vapor in the vials.

### 2.4. Data Analysis

PCA [3] is a projection method that allows an easy visualization of all the information contained in a dataset. In addition, PCA helps to find out in what respect a sample is different from others and which variables contribute the most to this difference. The data was standardized before carrying out the PCA, in order to avoid the effect of the different dimensions. The five main principal component values were extracted and then used as the input for the discrimination analysis. The choice of five principal components will be justified by the results (Table 1).

LDA is one of the most used classification procedures, which has proven successful in many applications. The method maximizes the variance between categories and minimizes the variance within categories.

The so-called back-propagation neural network (BPNN) [3] is one of the more promising future technologies in computing. The network processes the inputs and compares the resulting outputs against the desired outputs. Errors are then propagated back through the system, causing the system to adjust the weights that control the network. This process occurs over and over as the weights are continually tweaked. During the training of a network the same set of data is processed many times as the connection weights are refined.

## 3. Results and Discussion

### 3.1. The Extraction of the Original Feature Vector

**Figure 1.**
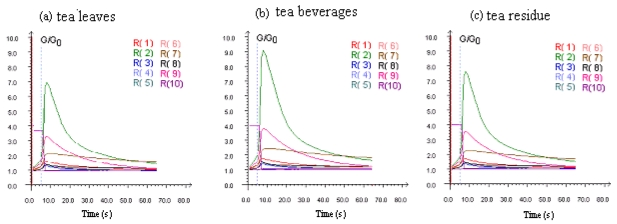
The response curve of tea leaves, tea brew and tea residue.

Figure 1 shows the typical responses of the E-nose for the tea leaves, tea beverages and tea residues. Each curve, marked R(1)… R(10), represents a different sensor transient that measures the conductivity of each sensor against time due to the electro-valve action when the volatiles reached the measurement chamber. In the initial period, the conductivity of each sensor increases rapidly, and reaches its maximum. Then the curve descends rapidly, and finally the change of the curve stabilizes after about 50 s. There were different response curve of each sensor for the tea leaves, tea beverages and tea residues.

In this research, response values that represent the different phases of the curve were investigated. The original feature vector consisted of eight different feature values of 10 sensors, that is:

$$X=(x_{1},x_{2},\ldots \ldots ,x_{80})$$

where *x*_1_–*x*_10_: response signals of each sensor at 7 s (presenting the ascending stage of the response curves),
*x*_11_……*x*_20_ : Response signals of each sensor at 15 s (presenting the rapidly descending stage of the response curve),
*x*_21_……*x*_30_ : Response signals of each sensor at 30 s (presenting the slowly descending stage of the response curve),
*x*_31_……*x*_40_ : Response signals of each sensor at 60 s (presenting the stable stage of the response curve),
*x*_41_……*x*_50_ : The maximum response signals of each sensor (the peak of each response curve),
*x*_51_……*x*_60_ : The average values of each response curve from the 45 s to 60 s,
*x*_61_……*x*_70_ : The integral values of each response curve in 60 seconds,
*x*_71_……*x*_80_ : The response signals with the maximum variance.

### 3.2. Principal Component Analysis (PCA)

The E-nose response for the tea contained in the original feature vectors [*X* = (*x*_1_, *x*_2_,…*x*_80_)] has too much information, and we can be sure that there is some information of little significance for the subsequent discrimination analysis. In order to improve the calculation efficiency and obtain a more efficient analysis, the dimensionality of the original feature vectors must be decreased. The information with little significance should be neglected and the useful information should be preserved. In the paper, to achieve this goal, PCA was carried out using *X* = (*x*_1_, *x*_2_,…*x*_80_).

Table 1 shows the eigenvalues and the accumulated contribution rate of the five main principal components of the tea leaf samples, tea beverage samples and tea residue samples. In this way, the dimensionality of the original feature vectors was decreased from 80 to 5. The results in Table 1 show that the accumulated contribution rate of the front five principal components was all more than 80%, most information of the original feature vectors was conserved, and then the five main principal components were used as the inputs for the discrimination analysis.

### 3.3. Linear Discrimination Analysis (LDA)

**Figure 2.**
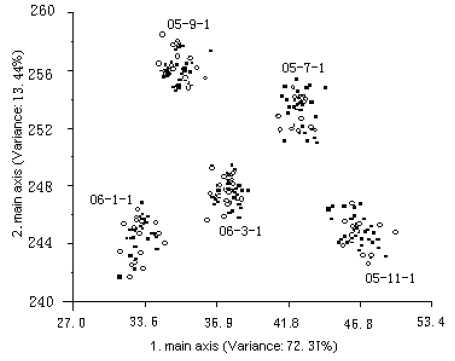
Discrimination and testing result of LDA based on the tea leaves.

**Figure 3.**
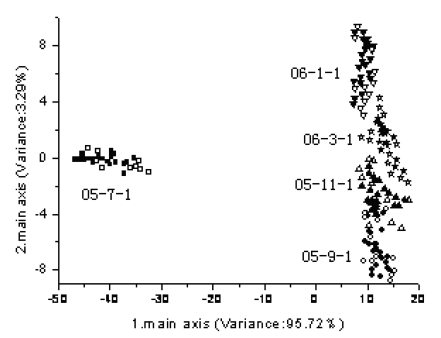
Discrimination and testing result of LDA based on the tea beverages.

**Figure 4.**
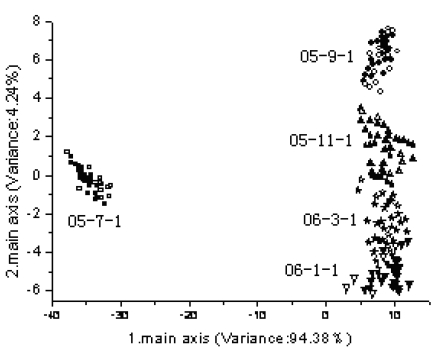
Discrimination and testing result of LDA based on the tea residues.

The five main principal components values are extracted by PCA and are analyzed by LDA. All the samples are separated into two groups. One group, the training set, contained 150 samples, and another is the test set which contained 75 samples. The analytical results of LDA based on tea leaves, tea beverages and tea residues are shown in Figures 2–4 (solid marks—training samples, hollow dots—test samples).

In Figure 2 better analytical results are obtained for both the training set and test set and the different storage times are obviously discriminated. This can be explained by the fact that there are more differences in the volatile component of the tea leaves with different storage times, which induces the responses of the sensors to change.

As shown in Figures 3–4, the analytical results on the basis of tea beverage or tea residues are worse than that on the basis of tea leaves. In Figure 3, the tea beverage samples for fresh tea are obviously separated, but the other samples are overlapped in a certain extent. In Figure 4, the analytical results based on the tea residue samples are similar to those based on tea beverage samples.

### 3.4. Back-Propagation Neural Network (BPNN)

In order to study the optimized discrimination method, a BPNN study was performed. The five main principal components acted as the inputs of the BPNN, and the output of the BPNN is the storage time (0, 60, 120, 180 and 240 days) of the tea samples. The network topology is 5-10-5, net.trainParam.lr = 0.08 and net.trainParam.lr_inc = 1.08. The software used is Matlab.

All the samples were also randomly separated into two groups. One group contained 150 samples (30 samples for each storage time) trained the BPNN and another group containing 75 samples (15 samples for each storage time) was tested. Test errors of the BPNN are recorded and these errors are calculated as:

$${\bar{X}}_{i}=\frac{1}{15}\sum_{j=1}^{15}\left|x_{i}-x_{ij}\right|$$

where *x*_i_ is the true storage time (*x*_i_ = 0, 60, 120, 180, 240), *x*_ij_ is the predicted storage time. The computed results are shown in Table 2.

As shown in Table 2, the *X̄_i_* is smaller for the tea leaves except for “0 day”, and the *X̄_i_* value is slightly higher when based on the tea beverages and tea residues. The BPNN results showed that the better prediction of the tea storage time was obtained based on the tea leaves using eigenvalues of the E-nose signals extracted by PCA.

## 4. Conclusions

First this study demonstrates the feasibility of using an E-nose as an analytical tool for the recognition of volatile components emitted by differently aged tea. Second it is feasible to obtain feature values extracted by a PCA method and then the five main principal components values are used as the input of the discrimination analysis. This method decreases the data dimensionality and optimizes the feature vector. The LDA results show that the classification result of the tea storage time was best when based on the tea leaves, and the BPNN results show that the predicted error is smaller when based on tea leaf samples than that on the basis of tea beverage samples and tea residue samples.

## Figures and Tables

**Table 1.** The eigenvalues and the accumulated contribution rate of the front five principal components of tea leaves, tea beverages and tea residues under different storage time.

	**No.**	**eigenvalues**	**Contribution rate %**	**Accumulated contribution rate %**
Tea leaf sample	1	49.25	48.93	48.93
	2	27.54	27.36	76.29
	3	11.25	11.18	87.47
	4	3.18	3.16	90.63
	5	2.27	2.26	92.89
Tea beverage sample	1	38.10	42.76	42.76
	2	18.34	20.58	63.34
	3	7.72	8.66	72.00
	4	4.86	5.45	77.45
	5	2.57	2.88	80.34
Tea residue sample	1	52.59	36.79	36.79
	2	29.01	20.29	57.08
	3	21.26	14.87	71.95
	4	9.47	6.62	78.57
	5	4.51	3.15	81.73

**Table 2.** The errors of the testing results.

*X̄_i_*	**0 (day)**	**60 (day)**	**120 (day)**	**180 (day)**	**240 (day)**
Tea leaves	9	2.73	3.93	6.33	6.8
Tea beverage	8	10.69	11.92	10.56	14.2
Tea residue	5.8	9.56	11.57	10.51	9.29
